# Pulmonary infection by *Rhodococcus equi* presenting with positive Ziehl-Neelsen stain in a patient with human immunodeficiency virus: a case report

**DOI:** 10.1186/1752-1947-8-423

**Published:** 2014-12-13

**Authors:** Anastasia Spiliopoulou, Stelios F Assimakopoulos, Antigoni Foka, Fevronia Kolonitsiou, Maria Lagadinou, Efthimia Petinaki, Evangelos D Anastassiou, Iris Spiliopoulou, Markos Marangos

**Affiliations:** 59 Department of Microbiology, University General Hospital of Patras, Ippokratous 1, Rion, 26504 Patras, Greece; 60 Division of Infectious Diseases, School of Medicine, University of Patras, Ippokratous 1, Rion, 26504 Patras, Greece; 61 Department of Microbiology, School of Medicine, University of Patras, Asklipeiou 1, Rion, 26504 Patras, Greece; 62 Department of Microbiology, School of Medicine, University of Thessaly, Biopolis, 41222 Larissa, Greece

**Keywords:** HIV, Pulmonary infection, Tuberculosis, *Rhodococcus equi*, Ziehl-Neelsen

## Abstract

**Introduction:**

Patients with human immunodeficiency virus carry a significant risk of contracting opportunistic infections. The worldwide increased incidence of tuberculosis has instituted pulmonary tuberculosis as an important diagnostic consideration in patients with human immunodeficiency virus presenting with lower respiratory tract infection. A positive result on the readily-available Ziehl-Neelsen stain usually leads to the initiation of antituberculous treatment, since tuberculosis may exert a rapid and even fatal clinical progress in human immunodeficiency virus coinfection. However, a number of other acid-fast bacteria might be implicated as offending pathogens. This case highlights the importance of broadening the list of pathogens that can account for a positive Ziehl-Neelsen stain in this select group of patients.

**Case presentation:**

We describe the case of a 34-year-old, Albanian man with untreated human immunodeficiency virus, presenting with clinical and radiologic signs of pulmonary tuberculosis and a positive Ziehl-Neelsen sputum specimen, who was finally diagnosed with pulmonary infection by *Rhodococcus equi.*

**Conclusions:**

*Rhodococcus equi* is a rare cause of pulmonary disease, even in patients with human immunodeficiency virus, and a positive Ziehl-Neelsen sputum specimen often misleads clinicians to more common organisms such as mycobacteria. A high index of suspicion, broadening the spectrum of optional pathogens, and effective communication between clinicians and microbiologists can ensure an efficient diagnostic and therapeutic approach.

## Introduction

Although opportunistic infections (OIs) in individuals with human immunodeficiency virus (HIV) have become rare in industrialized countries since the introduction of highly active antiretroviral therapy, patients continue to present with advanced HIV and HIV-related OIs [1]. Diagnosis of OIs in patients with HIV infection is a challenging medical task, in which effective clinical management requires firstly, a high index of clinical suspicion and secondly, the determination of the offending opportunistic pathogen, which will guide the institution of specific treatment in most cases.

Patients with HIV presenting with pulmonary infections may suffer from common bacterial pneumonias (60%), *Pneumocystis jirovecii*-associated pneumonia (20%), and mycobacterial pneumonias (20%), mostly caused by *Mycobacterium tuberculosis* (MTB), and less frequently by non-tuberculous mycobacteria (NTM), viral pneumonias (5%), fungal pneumonias (2%), parasitic pneumonias (0.5%), and multiple-pathogen pneumonia (7%) [2]. Intersection of the HIV and tuberculosis (TB) epidemics has led to a substantial increase in global TB incidence. In a compatible clinical course, pulmonary tuberculosis in a patient with HIV should be timely diagnosed and treated since it may exert a rapid and even fatal clinical progress [3].

In patients with HIV who are suspected of having TB by clinical and/or radiological criteria, the most important diagnostic tests are repeated sputum or bronchial samples for smear and culture [4]. In this patient population a positive smear for acid-fast bacilli (AFB), as detected by the Ziehl-Neelsen stain (Z-N), is very specific of MTB, even in a setting with a high incidence of *M. avium* complex that stains similarly [5]. Therefore, in such cases, initiation of antituberculous treatment is the usual practice while waiting upon specific culture results.

In this case report, we describe the case of a patient with untreated HIV who presented with clinical and radiological signs of pulmonary tuberculosis and a positive Z-N sputum specimen, who was finally diagnosed with pulmonary infection by *Rhodococcus equi*, an acid-fast, Gram-positive coccobacillus, which is increasingly recognized as an opportunistic pathogen in immunocompromised patients.

## Case presentation

**Figure 1:**
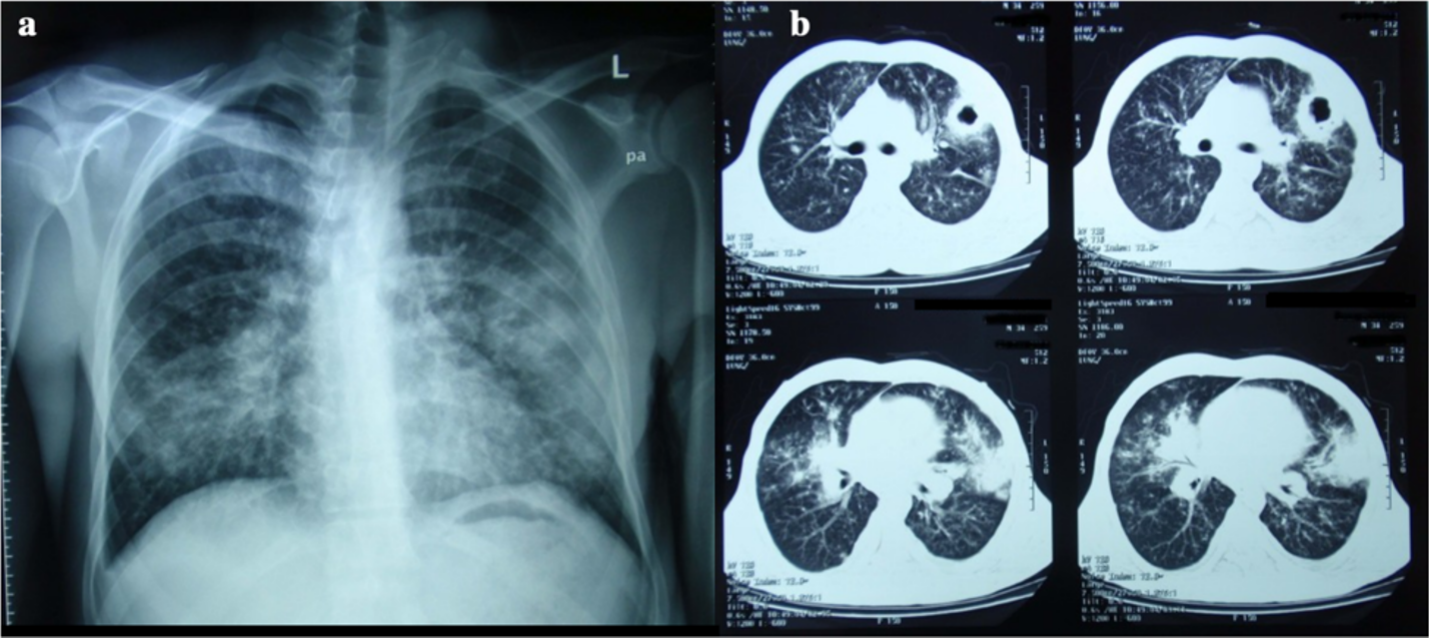
**Chest X-ray (a) and contrast-enhanced computed tomography scan (b) of our patient at admission demonstrating bilateral infiltrates located mainly at the lower lung lobes and a cavitary lesion at the lingula.**

A 34-year-old Albanian man was admitted to a regional hospital presenting with dry cough, fever, progressive dyspnea, night sweats, and a thick-walled cavitary lesion of the left lung, associated with bilateral pulmonary infiltrates on radiologic examination (Figure 1a and b). He had been well in other aspects until 15 days previous, except that he had lost 10kg in the last six months. While in the emergency department, he tested positive for HIV-1 infection and was referred to our hospital.

He had emigrated from Albania to Greece 10 years previously and worked as a farmer. He resided with his wife and two children, all of whom were well. There was no history of previous chest pain or cardiac disease, cough, sputum production, sweats, chills, use of tobacco or more than moderate amounts of alcohol, intravenous drug use, receipt of transfusions, rash, visual problems, headache, arthralgia, or myalgia. There was no history of recent travel, past tuberculous infection, or close contact with anyone with tuberculosis.

On admission, he was ill-looking and in considerable discomfort. His temperature was 38.35°C, his heart rate was 110 beats per minute, his respiratory rate was 30 breaths per minute, and his blood pressure 110/75mmHg. A physical examination revealed diffuse crackles in both lungs, while no friction rub was detected. There were also lesions consistent with Kaposi’s sarcoma on the oral mucous membrane and on his lower extremities. Multiple mobile lymph nodes of 1-cm diameter were palpated in both axillas, whilst no additional lymph nodes were felt. A specimen of arterial blood, drawn while he was breathing room air, disclosed that the partial pressure of oxygen (PaO2) was 77mmHg, the partial pressure of carbon dioxide (PaCO2) was 33mmHg, and the pH was 7.46. His hematocrit level was 33.0%, his hemoglobin level was 11g/dL (mean corpuscular volume was 85), his white cell count was 6.13×10^9^/L with 71.70% neutrophils, 16.50% lymphocytes, and 8.30% monocytes, and his platelet count was 150×10^9^/L. His erythrocyte sedimentation rate was 50mm per hour and his C-reactive protein level was 7.00mg/dL (normal limit <0.5mg/dL). The results of his other laboratory tests were normal except for low albumin. His CD4+ cell count was 4 cells/mm^3^ and his HIV-1 RNA was 71,045 copies/mL.

A microscopic examination of the stained sputum showed AFB and a cytologic examination revealed no malignant tumor cells. A culture of sputum specimens yielded normal respiratory tract flora. Specimens were subsequently inoculated onto Löwenstein-Jensen (L-J) medium. The initial viral serology tests performed on day two (including cytomegalovirus [CMV], coxsackie, Epstein-Barr virus, hepatitis A, B, and C viruses, herpes simplex virus [HSV] 1 and 2, and varicella zoster virus) were negative except from CMV immunoglobulin G (IgG). Based on these initial microbiological results, a possible diagnosis of MTB infection was presumed, and anti-TB treatment was initiated (isoniazid 5mg/kg/d, rifampicin 600mg/d, pyrazinamide 30mg/kg/d, and ethambutol 20mg/kg/d), in combination with empirical antimicrobial treatment for common bacterial pathogens (ceftriaxone 2gr/d and moxifloxacin 400mg/d) and prophylaxis with co-trimoxazole (1 tablet at 960mg, three times per week) and azithromycin (2 tablets totaling 1200mg/week). Five days later a marked increase of liver function was noted and the rifampicin was discontinued. He developed CMV antigenemia (pp65 positive) and therapy with ganciclovir (5mg/kg twice daily) was started. Subsequently, he developed neutropenia and his ganciclovir dosage was adjusted. Because of his low CD4 count, combined antiretroviral therapy (emtricitabine 600mg/d, tenofovir 200mg/d, and efavirenz 245mg/d) was introduced within two weeks from his admission. A repeated radiograph and thoracic computed tomography scan disclosed deterioration of pulmonary infiltrates, while he continued to be febrile. Real-time polymerase chain reaction (RT-PCR) (MTB COBAS® TaqMan® 48 Analyzer, Roche Diagnostics, Basel, Switzerland) performed directly in the clinical specimens was negative for the presence of MTB DNA.

**Figure 2:**
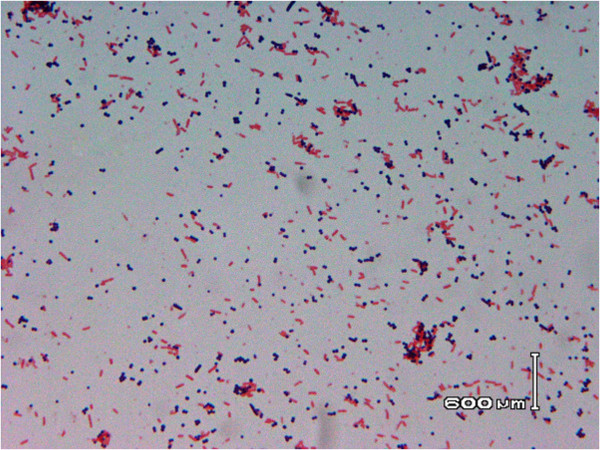
**Gram stain performed from positive blood culture vial revealing the presence of Gram-variable pleomorphic, coccoid, and bacillary bacteria.**

**Figure 3:**
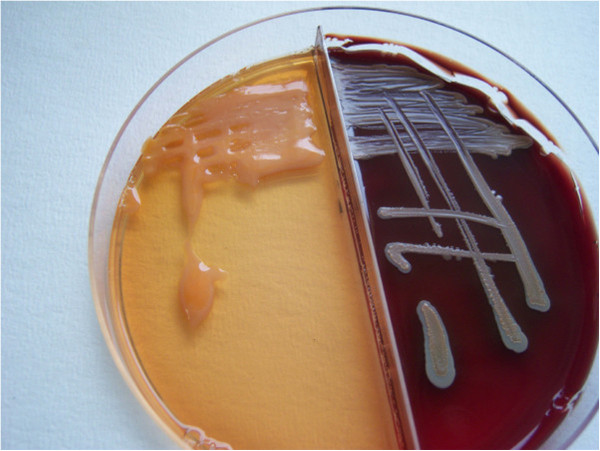
**Subculture of positive blood culture vial onto solid media.** Colonies are irregularly round, smooth, semitransparent, and mucoid, with a salmon-pink color that appeared on the fourth day of incubation at 30°C.

His respiratory status further deteriorated, but at this time aerobic vials from routine blood cultures (FA BacT/ALERT® and FN BacT/ALERT®, bioMérieux, Marcy l’Etoile, France) collected during his hospitalization showed as positive for microbial growth. Gram stain from the positive blood culture vials revealed the presence of Gram-variable pleomorphic, coccoid, and bacillary bacteria (Figure 2). After 24 hours of incubation onto MacConkey and blood agar plates, white, small colonies had grown. After prolonged incubation (over 48 hours), the colonies became smooth, glistening, and mucoid, had begun coalescing, and were teardrop-shaped, and after three to four days of incubation their color turned to salmon-pink (Figure 3). The isolated microorganism was catalase-positive and oxidase-negative. Phenotypic identification as *Rhodococcus equi* was initially performed by BBL GP cards (bionumber 1445000540, Becton Dickinson Diagnostics, Franklin Lakes, New Jersey, United States), whereas, by Vitek2 Advanced Expert System™ it was identified as *Kocuria varians* (bionumber 010010302000000, identification with 91% probability, bioMerieux). His urease test was positive, whereas, synergistic hemolysis was observed, with one hemolysin-producing *Staphylococcus aureus* strain. Susceptibility testing was performed by the disk diffusion method and Etest [6]. The isolate was susceptible to gentamicin, rifampicin, kanamycin, tetracycline, ciprofloxacin, vancomycin (minimum inhibitory concentration, MIC: 0.75μg/mL), teicoplanin (MIC: 0.5μg/mL), linezolid (MIC: 0.125μg/mL), imipenem (MIC: 0.19μg/mL), tigecycline (MIC: 0.094μg/mL), and daptomycin (MIC: 0.19μg/mL), and was resistant to ampicillin, cefoxitin, clindamycin, sulfamethoxazole/trimethoprim.

**Figure 4:**
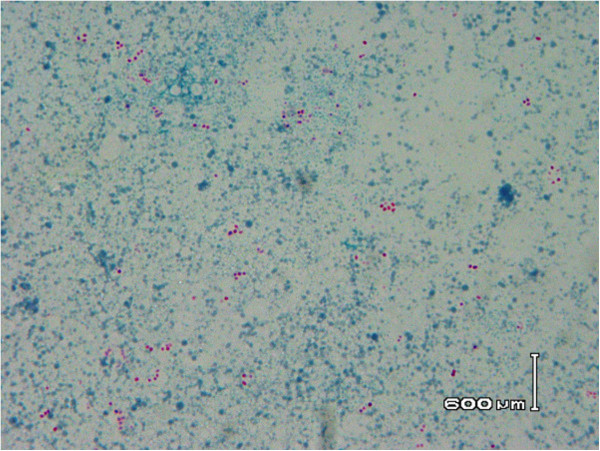
**Ziehl-Neelsen stain from the Löwenstein-Jensen medium inoculated with sputa samples.** The coccoid morphology of stained bacteria was not consistent with mycobacteria.

Identification to species level was confirmed performing PCR followed by sequencing of 23S rDNA gene and comparison with existing universal microbial genes’ sequencing data (BLAST database). Homology of 98% was detected with the *R. equi* genome, verifying the initial phenotypic identification. After diagnosis of *R. equi* bacteraemia, antibiotic treatment was adjusted and ertapenem combined with ciprofloxacin was administered intravenously. Three days later he became afebrile, with gradual improvement over the next fifteen days. Ten days after the identification of bacteraemia, growth was observed on the L-J medium inoculated with sputa. The Z-N stain was positive for AFB, but their coccoid morphology was not consistent with mycobacteria (Figure 4). A subculture on conventional solid media confirmed the presence of *R. equi*. Unfortunately, our patient died one month later due to CMV pneumonia.

## Discussion

*R. equi* (formerly *Corynebacterium equi*) was first isolated in 1923 from suppurative pulmonary lesions in foals [7]. The organism is present in soil in all continents except Antarctica, thrives in freshwater and marine habitats, and can live in the intestines of bloodsucking arthropods [8]. In the majority of cases, *R. equi* is acquired by inhalation or aerosols coming from the stool of infected foals, and a strong association with exposure to livestock or farming environments is referred [9]. The manifestations of *R. equi* infection are extremely diverse, although pulmonary infection is present in 80% of cases and may involve nodular infiltrates, pneumonic consolidation, pleural effusion, or empyema [8]. Spreading of the infection to other organs is common, particularly in immunocompromised subjects [10]. Bacteraemia occurs in over 80% of immunocompromised patients [11]. Respiratory disease tends to follow a subacute course with progressive cough, pleuritic chest pain, and fever often accompanied by cachexia, weight loss, and fatigue [12].

*R. equi* is an asporogenous, nonmotile, Gram-positive, obligate aerobe, growing optimally at 30°C but also at temperatures ranging from 10°C to 40°C. Colonies form on solid media in 48 hours or less and appear irregularly round, smooth, semitransparent, glistening, and mucoid. The characteristic salmon-pink color may not appear until days four to seven [13]. The organism varies from distinctly coccoid to bacillary, appearing coccoid on solid media or in purulent tissue, but forming long rods or short filaments with rudimentary branching in liquid media [9]. *R. equi* is characterized by the presence of catalase, urease, lipase, and phosphatase and by the absence of oxidase, DNAse, elastase, lecithinase, and protease [13]. Automated systems may lead to ambiguous results, as denoted in other studies [14]. Results of acid-fast staining are highly variable and *R. equi* can easily be mistaken for *Mycobacterium* organisms. In a reported case, a 56-year-old man who was HIV-seronegative was initially diagnosed with active pulmonary tuberculosis, and was treated with a multidrug antituberculosis regimen for three months despite a negative culture for mycobacteria [15]. Sputum smears were still positive for AFB and no clinical improvement was made until *R. equi* was isolated from the sputum [15]. Acid-fastness of *Rhodococcus*, along with clinical presentation and radiographic findings, lead to frequent misdiagnosis as pulmonary tuberculosis [9]. Besides *R. equi*, other respiratory pathogens share the acid-fast staining ability of MTB and NTM, and therefore, clinicians should be aware of the potential for misdiagnosis of these organisms as mycobacteria or the possibility of coinfection among those with persistently positive acid-fast staining smears.

There is no standard treatment for infection with *Rhodococcus* and treatment usually consists of a combination of at least two antibiotics to which the agent is susceptible [9]. These include macrolides, rifampicin, fluoroquinolones, aminoglycosides, glycopeptides, and carbapenems, although newer drugs, such as tigecycline and linezolid, have also been successfully used [10]. Our isolate was susceptible to rifampicin, ciprofloxacin, aminoglycosides, glycopeptides, and carbapenems, as expected, as well as tigecycline and linezolid, whereas resistance to beta-lactam antibiotics was observed as reported [9]. The choice should be based on the results of antibiotic susceptibility testing and drugs must be given intravenously for at least two weeks, followed by prolonged oral suppressive antibiotic treatment, whereas, surgical drainage of abscesses or cavitary lesions may also be required [10].

Despite treatment, the outcome of *Rhodococcus* infection is poor in immunocompromised patients, with the highest mortality (50 to 60%) in those with HIV coinfection [10]. The use of highly active antiretroviral therapy, however, has dramatically changed the prognosis in patients with HIV, with reported survival rates of virtually 100% [10].

## Conclusions

*R. equi* is a rare cause of pulmonary disease, even in patients with HIV, and the diagnosis may be problematic because of a low index of suspicion and frequent misidentification for more common organisms such as mycobacteria. A positive Z-N stain in the sputum specimen of an eligible patient should not preclude a TB diagnosis. Besides *R. equi*, *Nocardia* spp., and *Legionella micdadei* can also represent facultative pathogens, either alone or as co-offenders. Communication between clinicians and the microbiology laboratory can ensure a prompt diagnosis, early initiation of antimicrobial and antiretroviral treatment, and a favorable outcome.

## Consent

Written informed consent was obtained from the wife of the patient for publication of this case report and any accompanying images. A copy of the written consent is available for review by the Editor-in-Chief of this journal.

## Abbreviations

AFB: Acid fast bacteria
CMV: Cytomegalovirus
HIV: Human immunodeficiency virus
IgG: immunoglobulin G
L-J: Löwenstein-Jensen
MIC: minimum inhibitory concentration
MTB: *Mycobacterium tuberculosis*
NTM: Non-tuberculous mycobacteria
OI: Opportunistic infection
RT-PCR: real-time polymerase chain reaction
TB: Tuberculosis
ZN: Ziehl-Neelsen stain.
